# An Instrument-Assisted Coracoid Pain Test: An Exploratory Diagnostic Accuracy Study

**DOI:** 10.3390/ijerph191912735

**Published:** 2022-10-05

**Authors:** Michel GCAM Mertens, Mira Meeus, Louise Pieters, Mercè Balasch-Bernat, Lirios Dueñas, Olivier Verborgt, Filip Struyf

**Affiliations:** 1 Research Group MOVANT, Department of Rehabilitation Sciences and Physiotherapy (REVAKI), University of Antwerp, Universiteitsplein 1, 2610 Wilrijk, Belgium; 2 Department of Rehabilitation Sciences and Physiotherapy, Ghent University, C. Heymanslaan 10, 9000 Ghent, Belgium; 3 Physiotherapy in Motion, Multi-Specialty Research Group (PTinMOTION), Department of Physiotherapy, University of Valencia, C/Gasco Oliag 5, 46010 Valencia, Spain; 4 Department of Orthopedic Surgery and Traumatology, AZ Monica, Harmoniestraat 68, 2018 Antwerp, Belgium

**Keywords:** frozen shoulder, diagnostic test accuracy, coracoid pain test, diagnostic tests

## Abstract

The coracoid pain test (CPT) could contribute to the diagnosis of frozen shoulder (FS) with palpation. However, due to assessor performance these values might be unreliable. Therefore, the aim was to explore the diagnostic accuracy of an instrument-assisted CPT and two alternative approaches (pain severity and side comparison) for assistance in the diagnosis of FS. Patients with FS and healthy age-matched controls were recruited. All participants underwent the instrument-assisted CPT on both shoulders with a pressure algometer. Sensitivity, specificity, and likelihood ratios were determined for the three approaches. In total, 35 patients with FS and 35 healthy participants were included. The original approach was positive in eight participants (11.4%), with only sufficient specificity to draw a conclusion. The pain severity approach was positive in 31 participants (44.3%) with sufficient sensitivity, specificity and likelihood ratios. The side comparison approach was positive in 10 participants (14.3%) with excellent specificity and positive likelihood ratio. The specificity of the instrument-assisted CPT can be used to increase the probability of FS with both the original and alternative approaches. Only the pain severity approach can draw a conclusion with a negative test result. This study should be repeated with a cross-sectional design to strengthen and confirm the conclusions.

## 1. Introduction

Frozen shoulder (FS) is a condition characterized by progressive inflammation and fibrosis of the shoulder joint capsule and the rotator cuff interval, often resulting in severe pain and functional restriction of both active and passive shoulder motion [1,2]. There has been an increase in incidence and prevalence of FS over the last two decades [3], with a prevalence of 2–5.3% in the general population [4,5,6]. FS affects more women (up to 70%) than men [7,8,9] and usually develops between the age of 40 and 60 years [7,9]. Furthermore, the non-dominant shoulder is more affected than the dominant shoulder [7,9] and 6–34% of the patients develop FS on the contralateral shoulder [7,8,9]. In 14% of patients there is even a bilateral FS present [10].

However, FS remains a medical enigma, difficult to understand and difficult to manage. This also applies to the diagnosis of FS. Currently, diagnosis of FS in the early phase is mainly based on pattern recognition and clinical criteria. For this, clinical history taking, physical examination, and differential diagnosis are crucial [4,10,11,12]. Clinical diagnosis consists of confirming the characteristic pattern of mobility limitation [10] and exclusion of other pathologies, where necessary, with imaging [4,10]. Recently, Carbone et al. [13] analyzed the contribution of deep palpation on the coracoid area and concluded that palpation causing pain may be a pathognomonic sign of FS. During this specific test, termed coracoid pain test (CPT), Carbone et al. [13] applied pressure to the coracoid process, the acromioclavicular joint, and the anterolateral subacromial area. The participants were asked to score the severity of the pain on a visual analogue scale (VAS) from 0 (no pain) to 10 (most severe pain). The test was considered positive if the participants scored the pain three points higher on the coracoid process compared to the other two areas. They found almost excellent sensitivity and specificity (98% and 87%) and moderate to excellent positive and negative predictive values (61% to 99%) in the palpation over the coracoid process. In our opinion, there is an important limitation in the study of Carbone et al. [13], namely the pressure applied to the three areas might be unreliable. It could be that within each participant, a different pressure was applied to the three areas and the same could have happened between participants. Consequently, the results might be biased and more ideal results may have been achieved than what is actually correct. This could be improved by using an instrument to measure the pressure applied to each region.

Patients with FS experience a diffuse pain and a high pain intensity during the first phase in their affected shoulder, which might interfere with the original approach of the CPT. Therefore, pain severity and a comparison between the affected and unaffected shoulder might provide relevant diagnostic values as well. For the pain severity approach, the pain level of the three areas at the same shoulder can be used. The use of three classes for determining the severity of pain is suggested: mild (score: <3.4), moderate (score 3.5–7.4), and severe (score > 7.5) [14]. Therefore, we hypothesize that if pain at all three areas is scored moderate or severe, the diagnosis of FS might be more likely. For the side comparison approach, a difference between shoulders for each area was used. We hypothesize that with a difference larger than the minimally clinical important change of 1.1 point [15] for all three areas, the diagnosis of FS might be more likely.

In summary, the diagnosis of FS remains difficult; however, coracoid palpation seems promising as a contributing criterion for diagnosing FS during the painful phase in a population with frozen shoulder and healthy controls. Therefore, the aim of our study was to explore the diagnostic accuracy of three approaches (original, pain severity and side comparison) using an instrument-assisted CPT.

## 2. Materials and Methods

### 2.1. Study Design and Setting

This case-control diagnostic accuracy study (level III) was performed in the research laboratory of the department of Rehabilitation Sciences and Physiotherapy of the University of Antwerp. This study was part of a larger study concerning prognostic factors in patients with FS. The current study aimed to explore the sensitivity, specificity, and likelihood ratios of three different approaches (original, pain severity and side comparison) of an instrument-assisted CPT in patients with FS and healthy participants. Positive and negative predictive values are affected by prevalence [16,17], which makes them less useful in the current study because of the design used (and consequently the artificial constructed prevalence). For test selection, it is better to use the sensitivity, specificity, and likelihood ratios since they are unaffected by prevalence [17,18]. The ethics committee of University Hospital Antwerp approved the study (B300201422072). Written informed consent was obtained from all participants prior to commencement of the study.

### 2.2. Participants

Participants were recruited at the orthopedic department of AZ Monica, in general practitioner practices, and a sample of convenience or relatives of study participants between May 2018 and September 2020.

Eligibility criteria for all participants are presented in Table 1.

### 2.3. Procedure

First, all participants were asked to complete three questionnaires: a general questionnaire, the Disabilities of Arm, Shoulder, and Hand (DASH), and Shoulder Pain and Disability Index (SPADI) questionnaires. On physical examination, the instrument-assisted CPT was performed on both shoulders.

### 2.4. Questionnaires

#### General Questionnaire

A general questionnaire was used to acquire information about demographics, disease status and course, co-morbidities, work, and sports.

### 2.5. Disabilities of Arm, Shoulder, and Hand (DASH)

This questionnaire aims to assess symptoms and functional status (disability) in populations with upper extremity musculoskeletal conditions [20]. It comprises 30 items that address difficulty in performing physical activities requiring upper extremity function, symptoms of pain, activity-related pain, tingling, weakness, and stiffness. These items are scored on 5-item Likert scales, with a total score ranging from 0 (no disability) to 100 (most severe disability). The DASH was found valid and reliable in evaluating pain and disability in shoulder disorders [21].

### 2.6. Shoulder Pain and Disability Index (SPADI)

The SPADI is a self-reported index that consists of 13 items in two domains (pain (five items) and disability (eight items)) to measure pain and disability associated with shoulder disorders [22]. These 13 items are scored on a 0 to 10 scale, where 0 represents no pain or disability and 10 represents worst pain imaginable/so difficult as to require help. Each domain score is equally weighted and added to a total percentage ranging from 0 (no pain and disability) to 100 (worst pain and disability). The SPADI was found valid and reliable in evaluating pain and disability in shoulder disorders [21].

### 2.7. Instrument-Assisted Coracoid Pain Test (CPT)

**Figure 1:**
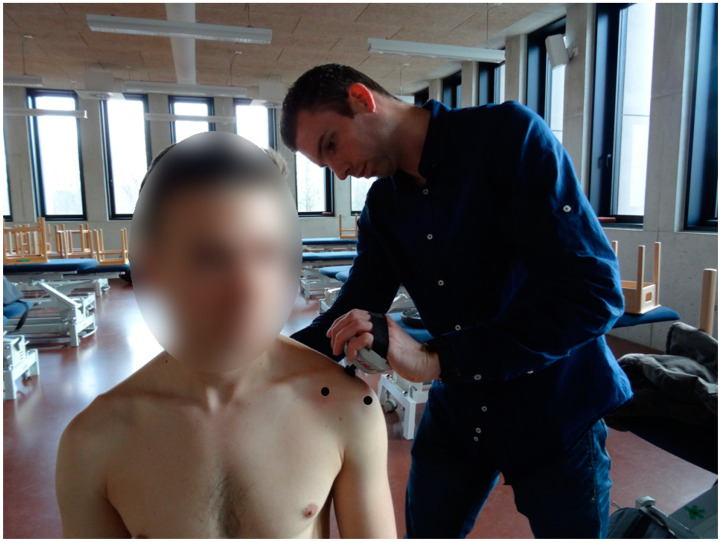
Pressure applied to the acromioclavicular joint, with the other areas marked with a black dot.

Patients were seated and the points of pressure (i.e., coracoid process, acromioclavicular (AC) joint, and the anterolateral subacromial area (two centimeters below the anterolateral angle of the acromion)) were marked with a pencil. During this test, pressure was built up gradually at 1 kg/cm^2^/second to 4 kg/cm^2^ with a digital pressure algometer (Wagner Instruments FDX 50, Greenwich, CT, USA) on these pressure points (Figure 1). This value was used in a previous study for palpation [23]. The participants scored the intensity of pain for each area on the verbal VAS from 0 (no pain) to 10 points (most severe pain). The test procedure was always first performed on the affected (or dominant) shoulder and followed by the unaffected (or non-dominant) shoulder. No familiarization trial on other regions was performed. The examination was performed by an experienced researcher (MM) with almost 10 years clinical experience. The original approach was considered positive when there was a difference in severity of at least three points between the coracoid process and both other two areas. The pain severity approach was considered positive if pain was rated at least moderate at all three areas. For the side comparison approach, the test was considered positive if there was a difference of at least 1.1 points on the VAS between sides for all three areas.

### 2.8. Statistics

Before the determination of the diagnostic values of the instrument-assisted CPT, the difference between group characteristics was checked with an independent T-test for continuous variables and Chi square or Fisher’s exact test for nominal variables.

The mean ± SD pain scores for each group, each area, and each side were determined and compared with an independent *t*-test. Within each group the results of the affected (dominant) and unaffected (non-dominant) side were compared with a *t*-test (normal distribution) or Mann–Whitney U test (no normal distribution). The level of significance was set at α < 0.05.

A 2 × 2 table with disease status and test result was created for the three approaches. The sensitivity, specificity, and positive and negative likelihood ratios with their 95%-confidence intervals (CI) were determined from these 2 × 2 tables. Likelihood ratios are more interesting from a clinical perspective since they provide a quantification of the probability of the disease given the results of the test [24]. With likelihood ratios, the best information is collected with very high values (positive likelihood ratio) and values closer to zero (negative likelihood ratio) [18]. With a high positive likelihood ratio the pre-test probability will shift to a higher post-test probability with a positive test result. With a small negative likelihood ratio, the pre-test probability will shift to a lower post-test probability with a negative test result. With values close to 1, the post-test probability will remain about the same for both positive and negative ratios. This pre-test probability will start between 2–5% (prevalence in the general population) and increase based on the findings in history taking and physical examination. The 95%-CI is used to indicate the preciseness of each value and is mandatory to allow for a critical evaluation of the estimates [18].

Statistical analysis was performed in R (version 3.6.2, Vienna, Austria). Diagnostic values were determined using add-on package epiR [25].

## 3. Results

### 3.1. Participants

**Figure 2:**
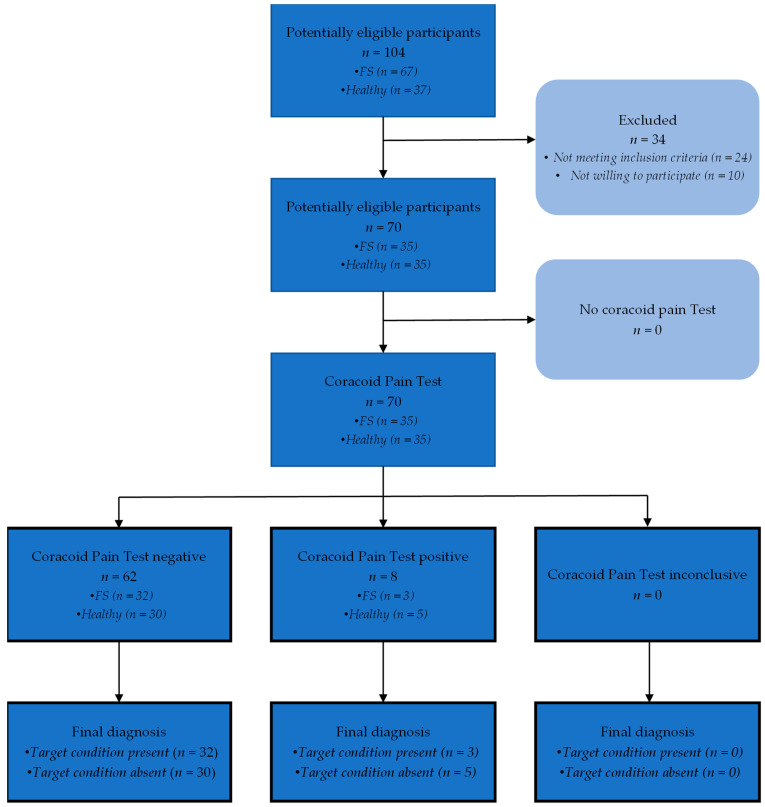
Participant flow of the study. FS: frozen shoulder.

Figure 2 shows the participant flow of the study. Finally, 70 participants were included (35 with FS and 35 healthy controls). All included patients with FS evolved towards FS. Table 2 shows the group characteristics of all included participants. There was a significant difference between groups for DASH, SPADI, and participation in sports (*p* < 0.01).

### 3.2. Pain Scores

Table 3 presents the mean pain scores for each region for both groups. There was a significant difference between both groups on both the affected and unaffected side for the pain score at the coracoid process, the AC joint, and the anterolateral subacromial area (*p* < 0.05). When the affected and unaffected side were compared within each group there was a significant difference between sides for all areas in the FS group (*p* < 0.05), but not in the healthy control group (*p* > 0.05).

### 3.3. Diagnostic Values

The mean diagnostic values of the three approaches are presented in Table 4.

#### 3.3.1. Original Approach

The instrument-assisted CPT was positive in eight of all 70 (11.4%) participants. In patients with FS, it was positive in three of 35 (8.6%), in healthy participants the test was positive in five of 35 (14.3%).

#### 3.3.2. Alternative Approach 1: Pain Severity

This approach was positive in 31 of all 70 (44.3%) participants. In patients with FS, it was positive in 25 of 35 (71.4%) and in healthy participants the test was positive in six of 35 (17.1%). The mean diagnostic values of the ‘pain severity’ approach are presented in Table 4.

#### 3.3.3. Alternative Approach 2: Comparison Affected–Unaffected Shoulder

This approach was positive in 10 of all 70 (14.3%) participants. The test was positive in 10 of 35 (28.6%) patients with FS, but not in the healthy group. The overall mean diagnostic values of the ‘within patient’ approach are presented in Table 4.

## 4. Discussion

The aim of the current study was to explore the diagnostic values of an instrument-assisted CPT in patients with painful phase FS. We found poor sensitivity, good specificity, and poor likelihood ratios with the original approach. The results show only small CI (±0.15) for sensitivity and specificity, other CIs are moderate to large (±0.17 to ±1.87), which indicates less precise results for the likelihood ratios.

With these results, the original approach of the instrument-assisted CPT can be used to rule in an FS with a positive test result (high specificity). However, the positive likelihood ratio is poor and cannot be used to improve the probability of the diagnosis FS. For this reason, we have to be careful with making strong conclusions. Furthermore, with a negative test result, we are unable to rule out the diagnosis FS, due to poor sensitivity and negative likelihood ratio.

Carbone et al. [13] were the only group to have investigated this diagnostic test so far. They found higher values for the sensitivity and specificity and much smaller CIs were reported. The difference in results might be a consequence of the procedure. Carbone et al. [13] used their fingers to provoke pain on the three different areas. Although an examiner might be experienced in palpating and provoking pain, there might be a difference in the pressure applied at the three areas within a patient and also between patients. Therefore, we used an algometer to apply the pressure, in this way the pressure was the same for all participants and should provide a more reliable measurement. With the use of an algometer, the performance of the test was identical for each participant and the pressure applied to each area was more reliable. Furthermore, the pressure used, is standardized and reliable as it is used in the diagnosis of fibromyalgia patients as well [23]. Therefore, the use of an algometer in the performance of the CPT is recommended and might explain the discrepancy with the study of Carbone et al. [13].

An explanation of the low number of positive tests might be the fact that patients with FS experience a diffuse pain in the shoulder as a consequence of more pronounced thickened, inflamed, and congested capsular joint [26]. This could explain the high pain score at the anterolateral subacromial area, the small difference between the pain scores at the coracoid process and the anterolateral subacromial area, and as a consequence a negative test result. Therefore, different approaches might be more beneficial for diagnosing FS in the painful phase. Another explanation might be the actual disease status of the included patients. Carbone et al. [13] did not provide any information about the disease stage of the included patients with FS, while in the current study only patients in the painful phase were included.

The suggested alternative approaches provided much better results compared to the original approach. When the pain severity is used as an alternative approach, the results indicate much better diagnostic values for both inclusion and exclusion of the diagnosis FS compared to the original approach and the 95% CIs are comparable. This might be explained by the diffuse pain experienced by the patients. Furthermore, when the difference between affected and unaffected shoulder is used as alternative approach, compared to the original approach, the diagnostic values for inclusion of the diagnosis FS are improved and the 95% CIs are comparable. Unfortunately, the values for ruling out FS are still not sufficient to draw a conclusion about the probability of the diagnosis. In addition, as the CPT might be more sensitive in the beginning with less diffuse pain, it might be interesting to investigate the CPT in different phases of the FS. Differences in test accuracy in the painful and stiff phase might provide additional information relevant for diagnosis and treatment guidance. In clinical practice, the instrument-assisted CPT can be performed as an additional assessment tool, which is convenient and easy applicable. With the original approach a positive test can increase the probability of the diagnosis of FS (sufficient specificity), but with a negative test result there is insufficient evidence to rule out the diagnosis of FS. When the pain severity approach is used, a positive test can be used to increase the probability of the diagnosis of FS (sufficient specificity and positive likelihood ratio) and a negative test can be used to decrease the probability of the diagnosis of FS (sufficient sensitivity and negative likelihood ratio). Finally, with the use of the affected-unaffected shoulder approach a positive test can be used to increase the probability of FS (excellent specificity and positive likelihood ratio), but with a negative test there is insufficient evidence to rule out FS. Beware that it is not recommended to use any of the approaches of the CPT as a stand-alone test, but rather it should be used as a supplement. Moreover, since these proposals are only based on the results in the current study be careful with interpretation based on these results. This should be investigated in future studies with higher methodological standards and the diagnostic value of these approaches should be confirmed.

In addition to the above suggestions, some other aspects of the CPT should be considered. Before large scale implementation and relying on the results of the CPT, repeatability, reproducibility and level of agreement should be investigated for example. When performing this test it is important to find similar results between different assessors, but also within the same assessor, to establish reliable results. Therefore, we recommend future studies to focus on these aspects of diagnostic accuracy as well.

### Limitations

The main limitation of the current study is the design. With this case-control design, a prevalence of FS of 50% was created, while the prevalence in the general population is 2–5%. Furthermore, only a comparison with healthy participants was made. To increase the strength of our conclusions, it is recommended to replicate this study with a cross-sectional design, including both patients with FS, other shoulder disorders and healthy participants.

Although the pressure elicited on the three areas can be standardized with an algometer, the area in which the pressure is applied is much smaller (1 cm^2^) compared to a finger and placement of the tool is therefore more difficult. In addition, when the finger applies the pressure, the intended area is simultaneously palpated and position can be corrected immediately, while with the algometer a correction is more difficult. On the other hand, when the position of the algometer is correct after palpation and marking the locations, the pressure can be elicited more reliably. Consequently, the test results will be more reliable.

Another limitation is the small sample size, although a sample size calculation usually is not performed for a diagnostic accuracy study, larger sample sizes are suggested [27]. In the current study, 70 participants were included, while for a reliable test result with a lower prevalence and smaller marginal error the sample size increases and numbers of hundreds or thousands of participants might be needed [27].

## 5. Conclusions

Although Carbone et al. [13] found almost excellent diagnostic values of the CPT; we were unable to reproduce these values with a more reliable method of applying the pressure to the different areas. Only a good specificity was found for this test with the original approach. With regard to pain severity approach sufficient specificity, sensitivity and likelihood ratios were found, while with the affected–unaffected shoulder approach resulted in excellent specificity and positive likelihood ratio. For future studies, a large sample size should be used, the use of an algometer for more reliable and standardized pressure is recommended and the likelihood ratios should be determined for appropriate use in clinical practice to assist in diagnosing FS in the painful phase with the instrument-assisted CPT. Furthermore, within the same studies the newly proposed approaches should be investigated as possible alternatives for the original approach. In the meantime, we advise caution with the use of this test, until confirmation of the results.

## Figures and Tables

**Table 1:** Eligibility criteria.

	Inclusion	Exclusion
Overall	Understand the Dutch language	Pregnant or breastfeeding women
Frozen shoulder	Stage 1 or 2 FS as defined by Hannafin & Chiaia [8]: duration of symptoms <9 months, pain with active and passive shoulder ROM & significant limitation of flexion, abduction, internal rotation, external rotation At least a 25% passive ROM deficit in two or more motion planes and 50% decreased in external rotation compared to the unaffected shoulder [4] Pain and movement restriction for at least 1 month and reached a plateau or deteriorating [4]	Shoulder complaints improved in the last month Shoulder surgery during the last year Locked dislocation Obvious symptoms of glenohumeral arthritis, fractures or avascular necrosis
Healthy controls	Matched for gender, age (±5 years), BMI, and hand dominance to the FS group	More than seven days with pain or discomfort in the neck-shoulder region during the past year and pain intensity on a NRS >2/10 [19] Pain or discomfort in more than three body regions for more than 30 days in the past year and pain intensity at the time of measurement on a NRS >2/10 [19] History of upper arm or shoulder fracture, shoulder surgery, or another shoulder pathology Neurologic disorders Systemic diseases (e.g., DM, rheumatoid arthritis) Cardiovascular diseases Glenohumeral osteoarthritis Depression or other psychiatric disorders Medication intake: pain killers or drugs influencing pain less than 48 h before the examination; opioids, anti-depressives and anti-epileptics less than two weeks before the examination; and use of corticosteroids or antihistamines

FS: frozen shoulder; ROM: range of motion; BMI: body mass index; NRS: numeric rating scale; DM: diabetes mellitus.

**Table 2:** Group characteristics of the included participants.

	Frozen Shoulder	Healthy
Age	53.40 ± 8.95	52.63 ± 6.93
Gender - Male - Female	12 (34.29%) 23 (65.71%)	14 (40.00%) 21 (60.00%)
Length	170.69 ± 7.27	172.80 ± 9.53
Weight	75.25 ± 15.21	72.26 ± 13.34
BMI	25.71 ± 4.24	24.07 ± 3.10
Hand dominance - Left - Right	6 (17.14%) 29 (82.86%)	6 (17.14%) 29 (82.86%)
Affected side - Left - Right - Bilateral	20 (57.14%) 13 (37.14%) 2 (5.71%)	Not applicable
Dominant side involved - Yes - No	14 (40.00%) 21 (60.00%)	Not applicable
Cause - primary - secondary	15 (42.86%) 20 (57.14%)	Not applicable
Diabetes mellitus - Yes - No	2 (5.71%) 33 (94.29%)	0 (0.00%) 35 (100.00%)
Thyroid disorder - Yes - No	6 (17.14%) 29 (82.86%)	0 (0.00%) 35 (100.00%)
**DASH**	49.63 ± 15.07	1.86 ± 3.04
**SPADI** - pain - disability - total	69.60 ± 16.70 65.14 ± 19.54 66.86 ± 17.15	0.40 ± 1.80 0.18 ± 0.87 0.26 ± 0.95
Work - no - Part time - Full time - Student	13 (38.24%) 8 (23.53%) 13 (38.24%) 0 (0.00%)	6 (17.14%) 10 (28.57%) 19 (54.29%) 0 (0.00%)
**Sport** - Yes - No	13 (39.39%) 20 (60.61%)	27 (79.41%) 7 (20.59%)
Sport - days - hours	3.30 ± 1.70 3.06 ± 2.44	3.29 ± 1.82 4.65 ± 3.27

BMI: body mass index; DASH: disabilities in arm shoulder and hand; SPADI: shoulder pain and disability index; Significant different variables between groups are in bold (*p* < 0.01).

**Table 3:** Pain (VAS) scores in mean ± SD in the three different areas for both groups.

Frozen Shoulder		Healthy		
	Affected	Unaffected	Dominant	Non-dominant
Coracoid process	7.36 ± 2.58	5.69 ± 2.93 *	3.94 ± 2.46	4.17 ± 2.43
AC joint	5.80 ± 2.96	3.94 ± 2.89 *	2.26 ± 2.24	2.11 ± 2.07
Anterolateral subacromial area	6.94 ± 2.89	4.57 ± 3.29 *	3.03 ± 2.46	2.77 ± 2.25

AC: acromioclavicular; * significant different between sides (*p* < 0.05).

**Table 4:** Diagnostic values for the original coracoid pain test.

	Value (95% Confidence Interval)		
	‘Original’ Approach	‘Pain Severity’ Approach	‘Within Patient’ Approach
**Sensitivity**	0.09 (0.02–0.23)	0.71 (0.54–0.85)	0.29 (0.15–0.46)
**Specificity**	0.86 (0.70–0.95)	0.83 (0.66–0.93)	1.00 (0.90–1.00)
**LR+**	0.60 (0.16–2.32)	4.17 (1.95–8.89)	∞
**LR−**	1.07 (0.90–1.26)	0.34 (0.20–0.59)	0.71 (0.58–0.88)

LR+: positive likelihood ratio; LR−: negative likelihood ratio.

## Data Availability

Data are available upon reasonable request from the corresponding author: filip.struyf@uantwerpen.be.
